# Pulmonary Hypertension: Unveiling Molecular Mechanisms, Diagnosis, and Therapeutic Targets

**DOI:** 10.3390/jpm13101446

**Published:** 2023-09-28

**Authors:** Marija Gredic, Stefan Hadzic

**Affiliations:** Excellence Cluster Cardiopulmonary Institute (CPI), University of Giessen and Marburg Lung Center (UGMLC), German Center for Lung Research (DZL), Justus-Liebig University, 35392 Giessen, Germany; marija.gredic@innere.med.uni-giessen.de

## 1. Introduction

Pulmonary hypertension (PH) is a progressive and life-threatening disease characterized by increased pulmonary arterial pressure, which leads to right heart hypertrophy and eventually right heart failure [1,2,3,4,5]. Although the pathogenesis of PH is not yet fully understood, contemporary research illuminated the intricate molecular mechanisms involved in developing this disease and spurred advancements in diagnostic precision, uncovering innovative therapeutic avenues [3,4,5]. In this editorial, we delve into the multifaceted nature of PH, exploring its diverse manifestations, molecular mechanisms, and advances in therapeutic strategies.

## 2. PH Diagnosis, Classification, and Epidemiology

PH is diagnosed via right heart catheterization, with mean pulmonary artery pressure (mPAP) > 20 mmHg at rest as the defining criterion. The distinction between pre-capillary PH (due to pulmonary vascular disease) and post-capillary PH (related to left heart disease) relies on pulmonary vascular resistance (PVR) and pulmonary artery wedge pressure (PAWP). A PVR ≥ 2 Wood units (WU) and PAWP ≤ 15 mmHg classify pre-capillary PH, whereas PAWP > 15 mmHg indicates post-capillary PH [1,3,6].

The estimated global prevalence of PH is approximately 1%. However, this prevalence is higher in older individuals due to the presence of cardiac and pulmonary causes of PH. According to the 2015 ESC/ERS Guidelines and the Proceedings of the 6th World Symposium on PH classification, PH patients can be classified into five groups: Group 1—pulmonary arterial hypertension (PAH); Group 2—related to left heart disease; Group 3—PH due to lung diseases and/or hypoxia; Group 4—chronic thromboembolic PH (CTEPH); and Group 5—a heterogeneous collection of PH syndromes [1,3,6].

PAH was more common in younger females but is now increasingly diagnosed in older patients, particularly those aged ≥ 65 years. Subtypes of PAH include idiopathic PAH (IPAH), heritable PAH, and drug- or toxin-associated PAH. Various drugs and toxins are associated with the development of PAH, with different levels of evidence supporting these associations [1,3].

Post-capillary PH, either isolated or combined with pre-capillary components, is common in heart failure (HF), particularly HF with preserved ejection fraction (HFpEF) and left-sided valvular diseases. Globally, left heart disease is the most common cause of PH, followed by lung diseases [3].

PH often accompanies advanced parenchymal and interstitial lung diseases, such as chronic obstructive pulmonary disease (COPD) and idiopathic pulmonary fibrosis [1,2,7,8,9,10]. Hypoxia, an environmental factor present at high altitudes, can lead to PH and chronic mountain sickness.

Chronic thromboembolic pulmonary hypertension (CTEPH) is on the rise due to increased awareness and screening. CTEPH incidence and prevalence vary but generally range from 2 to 6 and 26 to 38 cases per million adults, respectively [3,6].

Group 5 encompasses a complex group of disorders associated with PH. The exact incidence and prevalence of PH in these disorders are often unknown but can significantly impact morbidity and mortality. One example is PH associated with sarcoidosis [3,6].

## 3. Molecular Mechanisms in PAH

Familial pulmonary hypertension affects 3.2% of PH patients and involves the TGF-β family signaling pathway, particularly anaplastic lymphoma kinase (ALK)-1 and -5, bone morphogenetic protein receptor (BMPR)-2, endoglin receptor, and SMAD-8 mutations, which leads to abnormal vascular remodeling. BMPR-2 mutations, which are found in 55% of familial PAH and 26% of idiopathic cases, disrupt SMAD-1/-5/-8 signaling. BMP-4 also contributes to PAH via SMAD-dependent and independent pathways. Caveolin-1 gene (*CAV1*) mutations impact PAH by influencing signaling cascades, and *KCNK3* gene mutations impair the function of the K^+^ channel, both offering potential therapeutic targets. Eukaryotic translation initiation factor 2-alpha kinase 4 (*EIF2AK4*) gene mutations are linked to pulmonary veno-occlusive disease and pulmonary capillary hemangiomatosis, potentially explaining incomplete penetrance in BMPR2-related PAH [3].

Epigenetic modifications such as DNA methylation play a role in PAH. The hypermethylation of the BMPR-2 promoter in hereditary PAH can lead to a disease phenotype even in heterozygous mutant patients. Modifying BMPR-2 promoter methylation via drugs may be a therapeutic strategy. Another hypermethylated gene in PAH is ATP-binding cassette transporter A1 (ABCA1), which can be a biomarker for PAH risk [3,5,11].

Histone acetylation and deacetylation play a role in PAH regulation. Sirtuin (SIRT)-1 deficiency contributes to the proliferation of pulmonary artery smooth muscle cells (PASMCs) and pulmonary vascular remodeling. Resveratrol, a SIRT-1 activator, can counteract these effects. SIRT-1 also influences mitochondrial metabolism and glycolytic shifts via HIF-1α regulation [3,5,11].

The Src family of kinases and Rho-associated protein kinase (ROCK) are potential therapeutic targets in PAH [12]. Src kinases and their downstream effectors stabilize HIF-1α and HIF-2α, affecting prosurvival transcription factors [13]. ROCK inhibitors offer promise in reducing vasoconstriction and abnormal cell proliferation [14,15].

Metabolic reprogramming in endothelial cells, including glycolysis, fatty acid metabolism, and glutaminolysis, contributes to PAH development [16]. Dysfunctional mitochondria in PASMCs, characterized by mitochondrial fragmentation, may also play a role in PAH pathogenesis [17].

HIF-1α pathway activation in response to hypoxia leads to the transcriptional activation of genes involved in regulation of oxygen homeostasis. Elevated HIF-1α expression is observed in IPAH, even under normoxic conditions. Dysfunctional mitochondrial superoxide dismutase (MnSOD) in PAH creates a pseudohypoxic environment, leading to the activation of HIF-1α. ROS also stabilizes HIF-1α. HIF-1α affects NO metabolism, contributing to PAH severity. Understanding the HIF-1α pathway provides insights into potential therapeutic targets in PAH [18,19].

## 4. Therapeutic Approaches in Pulmonary Hypertension

Current FDA-approved therapies aim to address the imbalance between vasoactive and vasodilator mediators while restoring endothelial cell function. Traditional medications include prostacyclin analogs, phosphodiesterase (PDE)-5 inhibitors, endothelin receptor antagonists, and cGMP activators. These and other treatment advances, along with the use of combination therapies, resulted in marked improvements in patient outcomes. Nevertheless, PAH remains a fatal disease. Innovative therapeutic approaches have emerged, such as stem cell-based therapies, gene transfer, and epigenetic therapies, offering new hope for PAH management [3,15].

Researchers investigating the metabolic mechanisms underlying PAH have identified several potential therapeutic targets. Dichloroacetate (DCA), a pyruvate dehydrogenase kinase (PDK) inhibitor, has shown promise. Trimetazidine, another candidate drug, selectively inhibits fatty acid beta-oxidation, increasing glucose oxidation. It also restores intracellular phosphocreatine resources, optimizing cell metabolism and offering cytoprotective effects. Additionally, mitochondrial fission inhibition via mitochondrial division inhibitor 1 (Mdivi-1) reduces PASMC proliferation and restores ATP synthesis [15,17,19].

The cluster of differentiation (CD)-146/HIF-1α signaling pathway has emerged as a potential therapeutic target. CD146, found in the vascular wall, plays a role in PASMC differentiation, migration, and proliferation, intersecting with well-known signaling pathways relevant to PAH. Disrupting the CD146/HIF-1α axis limits PAH progression. Doxycycline and interferon 2α were explored as potential therapeutic options, and they have the ability to inhibit proliferation and collagen synthesis while modulating the immune system [18].

Dysregulated microRNAs (miRNAs) contribute to gene expression abnormalities in pulmonary vasculature, driving PAH pathogenesis. Restoring miRNA expression to normal levels offers a promising approach, especially for IPAH. Methods such as anti-miRNA (anti-miR) oligonucleotide-based and miRNA mimic-based approaches can be employed, focusing on targeted delivery to specific vascular cells and precise dosing to minimize off-target effects. Recent studies also suggest targeting dynamin-related protein (DRP)-1 adapter proteins and mitochondrial dynamic proteins MiD49 and MiD51 to reduce mitotic mitochondrial fission and promote the regression of pulmonary hypertension [3].

Kinases are emerging as potential drug targets in PAH management [4,12,20]. Rho kinase activation in PAH sensitizes calcium and increases vasoconstriction and PASMC proliferation, and inhibitors such as fasudil show short-term efficacy and safety in human PAH cohorts [21,22]. Imatinib, a tyrosine kinase inhibitor, may reverse PAH, but further trials are needed [12,20]. Targeting Src family kinases also holds therapeutic promise due to their involvement in ROS-induced vascular remodeling [5,6,13].

## 5. Conclusions

PH is a complex, multifactorial, and deadly disease. Advances in comprehending molecular mechanisms have led to the development of targeted therapies, rekindling hope for patients facing this diagnosis. Timely and precise diagnosis, guided by genetic and molecular insights, remains instrumental in enhancing the prognosis and quality of life for those living with PH. As research in the field continues to evolve, the future promises further breakthroughs in managing this complex and multifaceted disease, ultimately transforming the lives of countless individuals impacted by PH.
